# Commingling effect of gynoid and android fat patterns on cardiometabolic dysregulation in normal weight American adults

**DOI:** 10.1038/nutd.2015.5

**Published:** 2015-05-18

**Authors:** I S Okosun, J P Seale, R Lyn

**Affiliations:** 1 Division of Epidemiology & Biostatistics, School of Public Health, Georgia State University, Atlanta, GA, USA; 2 Department of Family Medicine, Medical Center of Central Georgia and Mercer University School of Medicine, Macon, GA, USA; 3 Division of Health Management & Policy, School of Public Health, Georgia State University, Atlanta, GA, USA

## Abstract

**Aim:**

To determine the independent and commingling effect of android and gynoid percent fat (measured using Dual Energy X-Ray Absorptiometry) on cardiometabolic dysregulation in normal weight American adults.

**Methods:**

The 2005–2006 data (*n*=1802) from the United States National Health and Nutritional Examination Surveys (NHANES) were used in this study. Associations of android percent fat, gynoid percent fat and their joint occurrence with risks of cardiometabolic risk factors were estimated using prevalence odds ratios from logistic regression analyses.

**Results:**

Android-gynoid percent fat ratio was more highly correlated with cardiometabolic dysregulation than android percent fat, gynoid percent fat or body mass index. Commingling of android and gynoid adiposities was associated with much greater odds of cardiometabolic risk factors than either android or gynoid adiposities. Commingling of android and gynoid adiposities was associated with 1.75 (95% confidence interval (CI)=1.42–2.93), 1.48 (95% CI=1.32–1.91), 1.61 (95% CI=1.50–1.89), 3.56 (95% CI=2.91–4.11) and 1.86 (95% CI=1.49–1.96) increased odds of elevated glucose, elevated blood pressure, elevated low-density lipoprotein-cholesterol, elevated triglyceride and low high-density lipoprotein-cholesterol, respectively.

**Conclusions:**

Normal weight subjects who present with both android and gynoid adiposities should be advised of the associated health risks. Both android and gynoid fat accumulations should be considered in developing public health strategies for reducing cardiometabolic disease risk in normal weight subjects.

## Introduction

Adiposity is a heterogeneous and multifaceted disorder in which subgroups of obese subjects present varying cardiometabolic profiles. Three of the well-known adiposity subgroups include metabolically healthy obese subjects, metabolically unhealthy obese subjects and metabolically healthy normal weight subjects. Metabolically healthy obese subjects have normal metabolic profiles despite elevated body fat.^1, 2^ Paradoxically, metabolically healthy obese subjects seem to be protected from metabolic disorders. Indeed, numerous studies have found that metabolically healthy obese subjects have high levels of insulin sensitivity and favorable lipids profiles as well as absence of dyslipidemia, diabetes and hypertension.^3, 4, 5, 6^ Metabolically unhealthy obese subjects, on the other hand, have clusters of cardiometabolic risk factors.^7^ Metabolically healthy normal weight subjects are characterized by normal metabolic profiles and normal body weight. Compared with metabolically healthy normal weight subjects, metabolically healthy obese subjects and metabolically unhealthy obese subjects have increased risk of developing type 2 diabetes, cardiovascular diseases and all-cause mortality.^8, 9^ Metabolically healthy normal weight subjects often do not present with clusters of metabolic and cardiovascular risk factors similar to what is often associated with being overweight or obese such as elevated fasting glucose, insulin resistance, increased triglyceride and decreased high-density lipoprotein (HDL)-cholesterol levels, and systemic inflammation.^10^ It is estimated that the metabolically unhealthy normal weight and metabolically healthy normal weight subgroups represent about 25% and 35% of the population, respectively.^10, 11^ Whereas many of the adiposity phenotypes are defined using body mass index (BMI), little is known about normal weight subjects who are abdominally obese and their risks for cardiometabolic risk factors.

Abdominal fat accumulation (defined using waist circumference) is a more potent correlate of cardiovascular diseases than generalized fat accumulation. The major advantage of waist circumference is the ease of measurement, but its major limitation in determining abdominal adiposity is that it does not take into account body build.^12, 13^ Hence, the International Society for Clinical Densitometry recommends Dual Energy X-Ray Absorptiometry (DEXA) for measuring abdominal adiposity.^14^ The advantage of DEXA includes speed, ease of use and low radiation exposure.^15, 16^ DEXA's ability to accurately and precisely measure body fat mass in various body compartments has been well validated.^17^ The joint occurrence of trunk (android) and hip (gynoid) fat accumulations that is independent of BMI is a yet to be well-described obesity phenotype. There are no available data regarding the association between DEXA-defined abdominal fat accumulation (elevated android percent fat) and cardiometabolic derangement in a sample of normal weight American adults. Using a more accurate measurement of site-specific body fat may provide a better understanding on the role of abdominal fat accumulation in cardiovascular diseases.

The aims of this study are to determine: (i) the association of DEXA-defined elevated android and gynoid percent fat with cardiometabolic risk factors, (ii) whether commingling of android and gynoid percent fat is associated with greater cardiometabolic deregulation than their independent effect in normal weight American adults.

## Methods and procedures

### Study design

The 2005–2006 data from the United States National Health and Nutritional Examination Surveys (NHANES) were used in this study. These surveys are based on cross-sectional sampling designs that collect health-related information from noninstitutionalized American adults. NHANES participants were interviewed in their homes and subsequently received physical and laboratory examinations in mobile examination centers. Detailed description of the NHANES methodologies has been published elsewhere,^18^ and is also available at the National Center for Health Statistics (NCHS) website.^19^ The surveys were based on stratified, multistage probability sampling technique. The stages of sample selection were as follows: (i) Primary Sampling Units were counties or small groups of contiguous counties; (ii) segments within Primary Sampling Units (a block or group of blocks containing a cluster of households); (iii) households within segments; and (iv) one or more participants within households.^18^ The institutional review board of NCHS approved the protocol for the NHANES.

### Study population

This study was restricted to normal weight (BMI<25) adults 18 years and older (*n*=1802) who were assayed using DEXA and with values for the following variables: sex, age, waist circumference, height, weight and fasting plasma glucose (FPG), oral glucose tolerance test, triglycerides, HDL-cholesterol, low-density lipoprotein-cholesterol (LDL-cholesterol), triglycerides, blood pressure (BP), and android and gynoid percent fat.

### Measures

In the survey that was used in this analysis, sex, age and race/ethnicity were determined as reported by participants. In NHANES, anthropometric measures and biological samples were obtained in mobile examination centers. Descriptions of variable measurements and assays are available online.^18^ Waist circumference was obtained using nonelastic tape and assessed above the right iliac crest at the mid-axillary line. Height was measured using a fixed stadiometer with a vertical backboard and a moveable headboard. Weight was measured at a standing position using a Toledo digital weight scale (Seritex, Carlstadt, NJ, USA), and measurement was made at the end of a normal expiration and to the nearest 0.1 kg. Three consecutive BP readings were obtained at a one-time examination visit using a standard protocol. In this investigation, averages of the three systolic (SBP) and diastolic BP (DBP) readings were used as representative of the participants' SBP and DBP values.

In NHANES, lipids (triglycerides, HDL-cholesterol and blood glucose) were measured after 8 h of an overnight fast. Triglycerides and glucose were measured enzymatically in serum using a series of coupled reactions after hydroxylation into glycerol. HDL-cholesterol measurements for the 2005–2006 surveys were attained using a direct immunoassay technique. The Fairview Medical Center Laboratory at the University of Minnesota performed glucose measurements using the Roche/Hitachi 911 Analyzer (Roche Diagnostics, Indianapolis, IN, USA). Fasting glucose was measured according to a hexokinase enzymatic method.^18, 19^ LDL-cholesterol was calculated using Friedewald's equation [LDL-cholesterol=total cholesterol-HDL-cholesterol-(1/5) triglycerides] if triglycerides were less than 400 mg dl^−1^.

In NHANES, entire body DEXA scans were administered in the mobile examination center and the Hologic APEX software was used in the scan analysis to define the android and gynoid regions. The android area is roughly the area around the waist between the mid-point of the lumbar spine and the top of the pelvis while the gynoid area lies roughly between the head of the femur and mid-thigh.^19^

### Definition of terms

Normal weight was defined as BMI value of less than 25 kg m^−^^2^ and greater than 18 kg m^−^^2^. Elevated triglyceride was defined as triglyceride of ⩾150 mg dl^−1^.^20^ Low HDL-cholesterol was defined as <40 mg dl^−1^ in males and <50 mg dl^−1^ in females.^20^ Elevated LDL-cholesterol was defined as 100 md dl^−1^ or greater. Elevated BP was defined as (i) SBP (mean SBP) ⩾130 or (ii) DBP (mean DBP) ⩾85 mm Hg or (iii) treatment for previously diagnosed hypertension or (iv) taking a BP medication.^20^ Elevated glucose was defined as fasting plasma value of 125 mg dl^−1^ or greater. In this study, smoking was categorized as smokers and nonsmokers, and moderate alcohol intake as consuming more than two alcoholic drinks per day for men and one drink per day for women.^21^

#### Elevated android and gynoid percent fat

Subjects were divided into sex-specific tertiles of android percent fat as follows: first tertile (>11.4% men, >19.9% women), second tertile (>18.3 men, >28.1% women) and third tertile (>27.1% men, >35.8% women). Tertiles of gynoid percent fat were also computed as follows: first tertile (>13.7% men, >32.3% women), second tertile (>19.7% men, >36.7% women) and third tertile (>25.5% men, >39.7% women). Subjects with in the third tertile of android and gynoid percent fat were regarded as having elevated android and gynoid fat, respectively. Joint occurrence of android and gynoid fat accumulation was determined as having android and gynoid percent fat of >27.1% and >25.5%, respectively, for men. The analogous values for women were >35.8% and >39.7%, respectively. Android-gynoid percent fat ratio was defined as android fat divided by gynoid fat. Android-gynoid percent fat ratio is a pattern of body fat distribution that is associated with an increased risk for metabolic syndrome in healthy adults.^22, 23^ Android-gynoid percent fat ratio is an important predictor metabolic and cardiovascular disease risk in normal weight as well as overweight and obese children.^24^

### Statistical analyses

All study analyses were conducted using SAS for Windows version 9.2 (SAS Institute, Inc., Cary, NC, USA) and SAS callable SUDAAN. To account for the unequal probability of selection, oversampling and nonresponse, the appropriate sample weights, strata and cluster variables were utilized. Descriptive statistics were performed using the survey frequency and survey means function in SAS. We assessed cardiovascular risk of elevated android and gynoid percent fat rates by clustering of cardiometabolic risk factors (two or more, three or more and four or more cardiometabolic risk factors that includes elevated glucose, elevated BP, elevated LDL-cholesterol, elevated triglycerides and low HDL-cholesterol).

Overall and sex-specific correlations of android, gynoid, android-gynoid percent fat and BMI with cardiometabolic risk factors were assessed using age-, smoking- and alcohol intake-adjusted Pearson's correlation methods. Independent associations between elevated android and gynoid percent fat, and their joint occurrence (independent variables) with cardiometabolic dysregulations (elevated glucose, elevated BP, elevated LDL-cholesterol, elevated triglycerides, low HDL-cholesterol) were assessed using odds ratios from multiple logistic regression models. In all the logistic regression models, statistical adjustments were made for age, smoking and alcohol intake, and *P*-values of <0.05 and 95% confidence intervals were used to establish statistical significance.

## Results

### Basic characteristics of studied population

The basic anthropometric and clinical characteristics of the studied population of normal weight men (*n*=1171) and women (*n*=685) are shown in Table 1. The studied population had BP, triglycerides, FPG, LDL-cholesterol, HDL-cholesterol and total cholesterol values that were within the National Cholesterol Education Program recommendations. Men had statistically significant higher values of body weight, waist circumference, BP and triglycerides, and presented with higher rates of smoking when compared with women (*P*<0.01). Women had statistically significant higher values of android and gynoid percent fat when compared with men (*P*<0.01). There were no significant gender differences for age, BMI, FPG, LDL-cholesterol, HDL-cholesterol and total cholesterol differences.

### Rates of cardiometabolic abnormality according to adiposity phenotype

**Figure 1:**
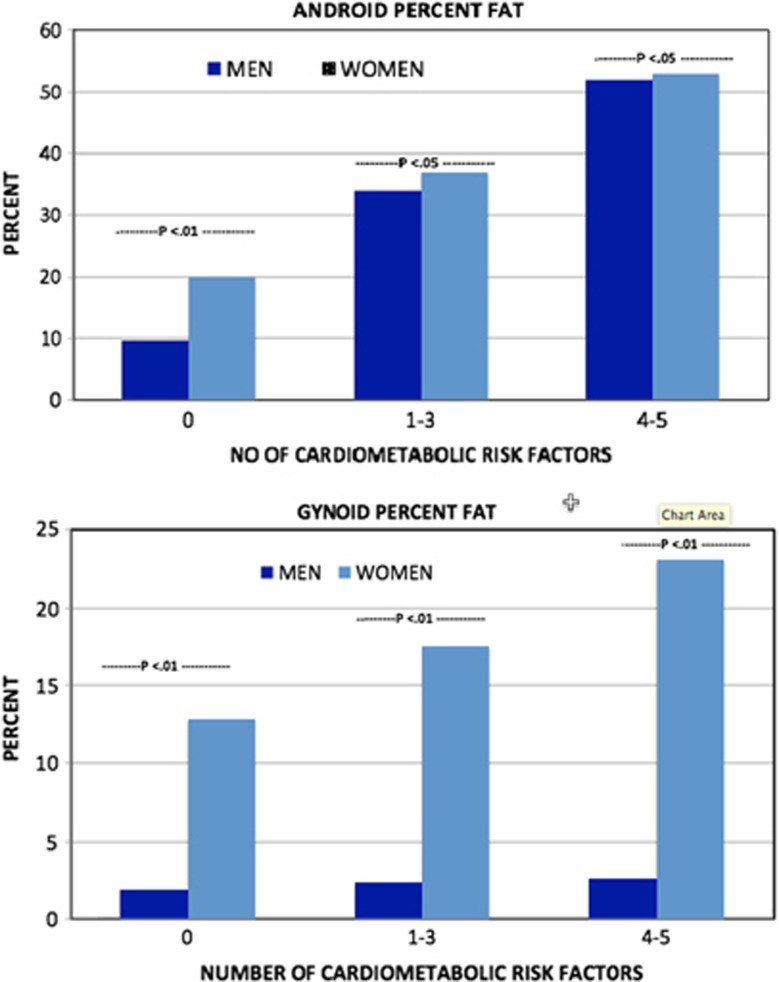
Prevalence of android and gynoid adiposity by numbers of cardiometabolic risk factors in non-overweight American adults.

Prevalences of android percent fat by the number of cardiometabolic risk factors were determined (Figure 1) using values >27.1% and >35.8%, for men and women, respectively, while gynoid percent fat was determined using values of >25.5% and >39.7%, for men and women, respectively. As shown, there were statistically significant gender differences in rates of android and gynoid percent fat at every level of cardiometabolic risk numbers. In men, the rate of android percent fat for subjects with 0, 1–3 and 4–5 cardiometabolic risk factors were 9.5%, 34% and 51.9% compared with 20%, 37% and 53%, for women, respectively. In men, the rate of gynoid percent fat for subjects with 0, 1–3 and 4–5 cardiometabolic risk factors were 1.9%, 2.3% and 2.6% compared to 12.8%, 17.6% and 23.1%, for women, respectively.

### Correlation analysis

We investigated age-, sex-, smoking- and alcohol intake-adjusted overall and sex-specific degrees of correlation of android percent fat, gynoid percent fat, android-gynoid percent fat ratio and BMI with cardiometabolic risk factors (Table 2). Overall, android percent fat was positively and significantly correlated with LDL-cholesterol (*r*=0.131), and negatively correlated with triglycerides (*r*=−0.113) and HDL-cholesterol (*r*=−0.183), after and adjusting for age, sex, smoking and alcohol intake. Overall, gynoid percent fat was positively and significantly correlated with triglycerides (*r*=−0.138), HDL-cholesterol (*r*=0.143) and negatively correlated with mean SBP (*r*=−0.183) and FPG (*r*=0.168). The degrees of correlation of android-gynoid percent fat ratio with cardiometabolic risk factors were higher than those between android percent fat or gynoid percent fat with cardiometabolic risk factors. Controlling for age, sex, smoking and alcohol intake, android-gynoid percent fat ratio was positively correlated with DBP (*r*=0.122), SBP (*r*=0.203), triglycerides (*r*=0.370), FPG (*r*=0.180), LDL-cholesterol (*r*=0.175) and total cholesterol (*r*=0.102) and negatively correlated with HDL-cholesterol (*r*=−0.384). Overall, BMI was less highly correlated with the cardiometabolic risk factors that were investigated compared with android-gynoid percent fat ratio. Controlling for age, sex, smoking and alcohol intake, BMI was positively correlated with mean SBP (*r*=0.136), triglycerides (*r*=0.251), LDL-cholesterol (*r*=0.170), and negatively correlated with HDL-cholesterol (*r*=−0.347).

In men, android percent fat was positively and significantly correlated with triglycerides (*r*=0.307), LDL-cholesterol (*r*=0.208) and total serum cholesterol (*r*=0.154), and negatively correlated with HDL-cholesterol (*r*=−0.344). Gynoid percent fat was positively and significantly correlated with triglycerides (*r*=0.169), LDL-cholesterol (*r*=0.197) and total serum cholesterol (*r*=0.164), and negatively correlated with HDL-cholesterol (*r*=−0.226). In men, android-gynoid percent fat ratio was positively correlated with mean DBP (*r*=0.112), mean SBP (*r*=0.115), triglycerides (*r*=0.281) and LDL-cholesterol (*r*=0.227), and negatively correlated with HDL-cholesterol (*r*=−0.384), controlling for age, smoking and alcohol intake. Similar to the results of the overall data, BMI was less positively correlated with triglycerides (*r*=0.244) and LDL-cholesterol (*r*=0.167), and negatively correlated with HDL-cholesterol (*r*=−0.278), controlling for age, sex, smoking and alcohol intake.

In women, android percent fat was positively correlated with triglycerides (*r*=0.224) and LDL-cholesterol (*r*=0.138), and negatively correlated with HDL-cholesterol (*r*=−0.251), while gynoid percent fat was negatively correlated with only HDL-cholesterol (*r*=−0.152). In women, android-gynoid percent fat ratio was positively correlated with triglycerides (*r*=0.329) and FPG (*r*=0.168), and negatively correlated with HDL-cholesterol (*r*=−0.219). Similar to males, BMI was also negatively correlated with HDL-cholesterol (*r*=−0.157), after controlling for age, sex, smoking and alcohol intake.

### Association of android and gynoid fat patterns with cardiometabolic dysregulation

Results of overall (Table 3) and sex-specific analyses (Tables 4 and 5) of association of android and gynoid fat patterns and their combined effects on cardiometabolic dysregulation, including elevated glucose, BP, LDL-cholesterol, triglycerides and low HDL-cholesterol were determined using age-, BMI-, smoking- and alcohol intake-adjusted logistic regression models. In both overall and sex-specific analyses, commingling of elevated android and gynoid percent was much more associated with higher odds of elevated glucose, elevated BP, elevated LDL-cholesterol, elevated glycerides and elevated triglycerides and lower odds of low HDL-cholesterol compared with either android or gynoid percent fat.

Overall (Table 3), and after adjusting for age, BMI, sex, smoking and alcohol intake, elevated android percent fat was associated with increased odds of elevated glucose (OR=1.31; 95% CI=1.12–2.93), elevated BP (OR=1.42; 95% CI=1.22–2.11), elevated LDL-cholesterol (OR=1.22; 95% CI=1.03–1.60), elevated triglycerides (OR=2.59; 95% CI=1.61–3.98) and low HDL-cholesterol (OR=1.79; 95% CI=1.47–1.89). Elevated gynoid percent fat was associated with decreased odds of elevated glucose (OR=0.87; 95% CI=0.72–0.93), elevated BP (OR=0.80; 95% CI=0.71–0.98), elevated triglycerides (OR=0.91; 95% CI=0.81–0.98) and low HDL-cholesterol (OR=0.81; 95% CI=0.52–0.91), controlling for age, sex, smoking and alcohol intake. Overall, commingling of elevated android and gynoid percent was associated with increased odds of elevated glucose (OR=1.75; 95% CI=1.42–2.93), elevated BP (OR=1.48; 95% CI=1.32–1.91), elevated LDL-cholesterol (OR=1.61; 95% CI=1.50–1.89), elevated triglycerides (OR=3.56; 95% CI=2.91–4.11) and low HDL-cholesterol (OR=1.86; 95% CI=1.49–1.96), adjusting for age, BMI, sex, smoking and alcohol intake.

In men (Table 4), elevated android percent fat was associated with increased odds of elevated BP (OR=1.21; 95% CI=1.06–1.77), elevated triglycerides (OR=2.41; 95% CI=2.03–5.75) and low HDL-cholesterol (OR=1.23; 95% CI=1.19–1.89), adjusting for age, BMI, smoking and alcohol intake. After adjusting for covariates, elevated gynoid percent fat was significantly associated with decreased odds of elevated LDL-cholesterol (OR=0.82; 95% CI=0.76–0.90), while commingling of android percent fat and gynoid percent fat was associated with increased odds of elevated glucose (OR=1.51; 95% CI=1.42–2.93), elevated BP (OR=1.48; 95% CI=1.32–1.91), elevated LDL-cholesterol (OR=1.55; 95% CI=1.30–1.99), elevated triglycerides (OR=2.47; 95% CI=2.21–3.98) and low HDL-cholesterol (OR=1.65; 95% CI=1.51–2.93).

In women (Table 5), after adjusting for age, BMI, smoking and alcohol intake, elevated android percent fat was only significantly associated with increased odds of low HDL-cholesterol (OR=1.52; 95% CI=1.29–2.93). After adjusting for covariates, elevated gynoid percent fat was significantly associated with decreased odds of elevated blood glucose (OR=0.87; 95% CI=0.72–0.93), elevated BPs (OR=0.82; 95% CI=0.71–0.99) and low HDL-cholesterol (OR=0.59; 95% CI=0.52–0.91), while commingling of android percent fat and gynoid percent fat was associated with increased odds of elevated glucose (OR=2.24; 95% CI=2.01–2.93), elevated BP (OR=1.82; 95% CI=1.62–1.91), elevated LDL-cholesterol (OR=1.77; 95% CI=1.50–1.89), elevated triglycerides (OR=3.56; 95% CI=2.61–3.98) and low HDL-cholesterol (OR=1.70; 95% CI=1.49–2.93).

## Discussion

Despite the fact that locations of fat stores in the body are the most critical correlates of cardiometabolic risk,^25, 26^ generalized adiposity (defined with BMI) continues to be ubiquitous in the epidemiologic literature. Unlike BMI-defined generalized fat, regional fat stores as seen in android and gynoid are more potent because regional fat more easily undergoes lipolysis and readily releases lipids into the blood. Android adiposity is characterized by intra-abdominal (visceral) fat and is associated with increased risk of cardiovascular disease, hypertension, hyperlipidemia, insulin resistance and type 2 diabetes.^27, 28^ Gynoid adiposity is characterized by large amounts of subcutaneous fat and more common in females than men, but is less associated with cardiometabolic risk compared with android adiposity.^29^

Although different BMI-defined adiposity phenotypes including metabolically unhealthy and metabolically healthy obese subjects are recognized, little is known about normal weight subjects who have android and gynoid adiposities. Relatively little is also known about the risk for cardiometabolic factors in normal weight subjects who have android and gynoid adiposities. Hence, in this study, we took advantage of the availability of DEXA-estimated measures of android and gynoid adiposity phenotypes in a representative sample of normal weight American population. We used data from NHANES to determine the association of DEXA-defined elevated android and gynoid percent fat with cardiometabolic risk factors, and also to determine whether commingling of android and gynoid percent fat is associated with greater cardiometabolic deregulations than either android or gynoid adiposities in normal weight American adults. Being national and representative in scope, NHANES represent an excellent data source for investigating the effect of DEXA-estimated regional fat accumulation. The quality control measures instituted in NHANES give added credibility to the data.

### The main findings

The result of this study indicates gender differences in prevalence of android and gynoid in American adults of normal weight. Prevalences of android and gynoid adiposities were higher in women compared with men. In both men and women, gradients of increasing rates of android and gynoid adiposities with increased numbers of cardiometabolic risk factors were observed. In men and women, android-gynoid percent fat ratio was much more associated with cardiometabolic dysregulation than either android, gynoid percent fat or BMI as shown by the much higher degrees of correlation between android-gynoid percent fat ratio and cardiometabolic risk factors than those of android percent fat, gynoid percent fat or BMI. In men, increase in android percent fat was correlated with increased values of triglycerides, LDL-cholesterol and total cholesterol and decreased value of HDL-cholesterol (*P*<0.05). In women, increase in android percent fat was correlated with increased values of triglycerides, LDL-cholesterol and decreased value of HDL-cholesterol (*P*<0.01). In men, gynoid percent fat was correlated with increased values of LDL-C and total cholesterol (*P*<0.01). In men, android-gynoid percent fat ratio was correlated with increased value of SBP and DBP, triglycerides and LDL-cholesterol, and negatively correlated with HDL-cholesterol after controlling for age, smoking and alcohol intake (*P*<0.05), while in women, android-gynoid percent fat was positively correlated with triglycerides and negatively correlated with HDL-cholesterol (*P*<0.05).

This study also showed gender differences in the response of gynoid percent fat and joint occurrence of android elevated percent fat and gynoid percent fat for cardiometabolic risk factors that included elevated glucose, BP, LDL-cholesterol, triglycerides and low HDL-cholesterol. In men, elevated android percent fat (being in the highest tertile) was associated with 21% increased odds of elevated BP, 141% elevated triglycerides and 23% low HDL-cholesterol, controlling for age, BMI, smoking and alcohol intake. Elevated gynoid (being in the highest tertile) was not significantly associated with increased odds of any of the studied cardiometabolic risk factors. Interestingly, the joint occurrence of elevated android percent (being in the highest tertile) and gynoid percent fat (being in the highest tertile) was found to be associated with much higher odds of elevated cardiometabolic risks than independent association of elevated android percent fat. The joint occurrence of android and gynoid percent fat was associated with 60%, 19%, 48%, 2% and 25% much greater odds for elevated glucose, BP, LDL-cholesterol, triglycerides and low HDL-cholesterol, respectively, than the odds that is associated with having only elevated android percent fat. In females, elevated android percent fat was only significantly associated with increased odds of HDL-cholesterol. However, elevated gynoid percent fat was associated with 13%, 18% and 41% decreased odds of elevated glucose, elevated BP and elevated low HDL-cholesterol, respectively. Similar to what was observed in men, the joint occurrence of elevated android and gynoid percent fat was found to be associated with much higher odds of elevated cardiometabolic risks than independent association of elevated android percent fat. The joint occurrence of android and gynoid percent fat was associated with 61%, 55%, 48%, 75% and 65% much greater odds for elevated glucose, BP, LDL-cholesterol, triglycerides and low HDL-cholesterol, respectively, than the odds that is associated with having only elevated gynoid percent fat.

Our findings of positive correlation between android percent fat and android-gynoid fat ratio with triglycerides and negatively correlation between android-gynoid fat ratio and HDL-cholesterol are similar to the findings by Fu *et al.*,^30^ in 18–79-year-old Chinese women. Like the result of this study, Fu *et al.*^30^ also found android percent fat and android-gynoid fat ratio to be significantly associated with decreased odds of HDL-cholesterol. Our finding is also in agreement with a study by De Larochellière *et al.*^31^ in nonobese and apparently healthy young women and men. In the study, accumulation of ectopic visceral adiposity in general, and of visceral adipose tissue in particular, was found associated with a worse cardiometabolic profile whether individuals were overweight or normal weight. Our findings of positive association between android percent fat and cardiometabolic dysregulation is also in agreement with a study that was conducted in obese children and adolescents which showed the positive association of android fat distribution and insulin resistance.^32^ The present study showed that android percent fat was much more positively associated with some cardiometabolic risk factors in men. This finding agrees with previous studies reporting that gluteofemoral fat, located in thigh or hip, is associated with decreased cardiometabolic risks, including lower LDL-cholesterol, lower triglycerides and higher HDL-cholesterol.^33^ Our findings of much increased rate of android percent fat with 0, 1–3 and 4–5 cardiometabolic risk factors in both men and women compared with gynoid percent fat is unclear, but may be due to the much higher correlation between android fat mass and visceral adipose tissue compared with the correlation between gynoid fat mass and visceral adipose tissue.^24, 25, 34^

### Limitations

Some limitations must be taken into account in the interpretation of results from this study. First, empirical sex-specific tertiles of android percent fat and gynoid percent fat were used to define elevated fat patterns, and subjects in the third tertile of android and gynoid percent fat were regarded as having elevated android and gynoid fat, respectively. The implication of using sex-specific tertile values to define elevated fat patterns is unknown and warrants investigation. Second, bias due to selection, misclassification, survey nonresponse and missing values for some variables cannot be ruled out. However, previous studies based on data from National Health and Nutrition Examination Surveys have shown little bias due to survey nonresponse.^35^ Third, as a cross-section study, directionality of the associations between dependent variables and the independent variables cannot be clearly established. Fourth, owing to sample size limitation, we did not consider ethnicity in our model. Ethnic differences and impact of race/ethnic differences in the association between regional fat accumulation and cardiometabolic risks are well known.^36^ Hence, one must be cognizant of limits of generalizability of the results from this study to other groups and other countries, as well as the limitations of statistical modeling techniques that were used.

## Conclusion

Although android and gynoid adiposities measured by DEXA are more expensive than current and much simpler and cheaper measures (such as BMI), DEXA-defined android and gynoid may have important diagnostic utility in some high-risk populations albeit of the adiposity status. Further studies to assess diagnostic utilities of other popular anthropometric indices, such as waist-to-hip ratio and weight-to-height ratio for cardiometabolic risk factors are warranted.

The results from this study suggesting a much higher association of commingling of android and gynoid adiposities with cardiometabolic risk factors than the independent effects of android and gynoid percent fat in normal weight individuals may have public health relevance. Both android and gynoid fat accumulations should be considered in developing public health strategies for reducing cardiometabolic disease risk in normal weight subjects. Normal weight subjects who present with joint occurrence of android and gynoid adiposities should be advised of the associated health risks such as cardiovascular disease and metabolic syndrome.

## Figures and Tables

**Table 1:** Basic anthropometric and clinical characteristics of eligible subjects

*Variable*	*All (*n=*1802)*	*Men (*n=*1117)*	*Women (*n=*685)*	P*-value*
Age (year)	34.9±15.4	34.7±15.9	35.1±15.0	0.103
Weight (kg)	62.9±9.7	68.7±8.4	57.1±7.0	<0.001
Waist circumference (cm)	79.9±7.1	82.4±7.8	77.4±5.8	<0.001
Body mass index (kg m^−^^2^)	22.0 ±2.0	22.3±2.0	21.7±2.1	0.128
Diastolic blood pressure (mmHg)	67.6±11.1	67.6±12.0	67.7±10.3	<0.001
Systolic blood pressure (mmHg)	115.7±15.1	118.8±14.3	112.8±15.2	<0.001
Triglycerides (mg dl^−1^)	95.2±59.3	102.2±66.0	86.8±48.7	0.014
Fasting plasma glucose (mg dl^−1^)	97.4±32.4	100.0±33.7	94.3±28.8	0.431
LDL-cholesterol (mg dl^−1^)	105.2±32.7	105.8±32.8	104.5±32.6	0.393
HDL-cholesterol (mg dl^−1^)	59.7±16.3	55.7±15.5	63.9±16.0	0.131
Total cholesterol (mg dl^−1^)	183.3±38.4	181.1±38.9	185.5±37.8	0.399
Android percent fat	23.0±8.1	18.8±6.9	27.2±7.0	<0.001
Gynoid percent fat	27.7±9.7	16.9±5.3	36.0±5.1	<0.001
Smoking (%)	36.9	47.7	25.9	<0.001
Alcohol intake (%)	14.4	14.1	14.6	0.835

Abbreviations: HDL-cholesterol, high-density lipoprotein-cholesterol; LDL-cholesterol, low-density lipoprotein cholesterol.

**Table 2:** Partial correlations between android percent fat, gynoid percent fat, android-gynoid percent fat ratio and BMI with cardiometabolic risk factors

*Variables*	*Android % fat*	*Gynoid % fat*	*Android-gynoid % fat ratio*	*Body mass index*
*Overall*				
MDBP	0.020	0.070	0.122**	0.046
MSBP	−0.022	−0.183**	203**	0.136**
TG	−0.113**	0.138**	0.370**	0.251**
FPG	0.033	−0.168**	0.180**	0.028
HDL-cholesterol	−0.183**	0.143**	−0.384**	−0.347**
LDL-cholesterol	0.131**	−0.025	0.175*	170**
TChol	0.090	−0.001	0.102*	0.072
				
*Men*				
MDBP	0.074	0.009	0.112*	0.072
MSBP	0.063	0.009	0.115*	0.094
TG	0.307*	0.169**	0.281**	0.244**
FPG	−0.033	−0.096	0.090	−0.064
HDL-cholesterol	−0.344**	−0.226**	−0.349**	−0.278**
LDL-cholesterol	0.208**	0.197**	0.227**	0.167**
TChol	0.154**	0.164**	0.058	0.102
				
*Women*				
MDBP	−0.002	−0.054	0.037	0.077
MSBP	0.079	−0.017	0.096	0.045
TG	0.224**	0.009	0.329**	0.065
FPG	0.081	−0.034	0.168*	0.057
HDL-cholesterol	−0.251**	−0.152*	−0.219*	−0.157*
LDL-cholesterol	0.138**	0.082	0.137	0.008
TChol	0.064	0.004	0.106	0.048

Abbreviations: FBG, fasting plasma glucose; HDL-cholesterol, high-density lipoprotein-cholesterol; LDL-cholesterol, low-density lipoprotein-cholesterol; MDBP, mean diastolic blood pressure; MSBP, mean systolic blood pressure; TChol, Total serum cholesterol; TG, triglyceride. **P<0.01; *P<0.05.

**Table 3:** Associations between android percent fat, gynoid percent fat and their joint occurrence on cardiometabolic deregulations

*Independent variable*	*Cardiometabolic risk factors*				
	*Elevated glucose*	*Elevated BP*	*Elevated LDL-C*	*Elevated triglycerides*	*Low HDL-C*
	*OR (95% CI)*	*OR (95% CI)*	*OR (95% CI)*	*OR (95% CI)*	*OR (95% CI)*
EAF	1.31 (1.12–2.93)	1.42 (1.22–2.11)	1.22 (1.03–1.60)	2.59 (1.61–3.98)	1.79 (1.47–1.89)
Age	1.09 (1.05–1.07)	1.06 (1.05–1.07)	1.04 (1.03–1.06)	1.01 (0.99–1.02)	1.02 (1.01–1.03)
BMI	1.14 (0.98–1.35)	1.09 (1.04–1.15)	1.11 (1.05–1.19)	1.18 (1.10–1.27)	0.89 (0.85–0.94)
Sex^a^	1.31 (0.37–4.58)	1.40 (0.98–2.01)	1.33 (0.85–2.08)	3.41 (1.62–7.17)	0.59 (0.38–0.92)
Smoking	1.26 (0.53–3.00)	1.37 (1.05–1.77)	0.88 (0.68–1.39)	1.54 (1.00–2.36)	0.86 (0.64–1.16)
Alcohol	2.25 (2.01–5.31)	1.09 (0.78–1.52)	0.87 (0.52–1.45)	0.82 (0.46–1.47)	0.57 (0.39–0.82)
EGF	0.87 (0.72–0.93)	0.80 (0.71–0.98)	0.78 (0.73–1.10)	0.91 (0.81–0.98)	0.81 (0.52–0.91)
Age	1.09 (1.05–1.12)	1.06 (1.05–1.08)	1.05 (1.03–1.06)	1.01 (0.99–1.02)	1.01 (1.01–1.04)
BMI	1.15 (1.09–1.35)	1.07 (1.04–1.15)	1.11 (1.05–1.19)	1.22 (1.10–1.27)	0.86 (0.81–0.97)
Sex^a^	0.36 (0.25–2.49)	1.72 (0.86–3.45)	0.80 (0.34–1.90)	1.74 (0.41–3.77)	0.65 (0.21–1.54)
Smoking	1.17 (1.03–3.00)	1.31 (1.11–1.79)	0.90 (0.68–1.39)	1.19 (1.00–2.36)	0.86 (0.61–1.21)
Alcohol	2.21 (1.41–5.31)	1.07 (1.05–1.50)	0.86 (0.52–1.45)	0.81 (0.46–1.47)	0.56 (0.44–0.92)
EAF and EGF	1.75 (1.42–2.93)	1.48 (1.32–1.91)	1.61 (1.50–1.89)	3.56 (2.91–4.11)	1.86 (1.49–1.96)
Age	1.09 (1.05–1.09)	1.06 (1.04–1.08)	1.05 (1.03–1.06)	1.01 (0.99–1.02)	1.02 (1.01–1.03)
BMI	1.17 (1.08–1.35)	1.08 (1.05–1.21)	1.12 (1.05–1.20)	1.26 (1.17–1.37)	0.87 (0.83–0.95)
Sex^a^	1.08 (0.28–4.20)	1.44 (1.00–2.15)	1.50 (0.93–2.40)	3.67 (1.58–8.65)	0.66 (0.41–1.07)
Smoking	1.22 (1.13–3.00)	1.35 (1.15–1.88)	0.95 (0.68–1.39)	1.25 (1.18–2.36)	0.87 (0.74–1.26)
Alcohol	2.19 (1.91–5.31)	1.07 (1.01–1.52)	0.86 (0.52–1.45)	0.80 (0.46–1.47)	0.56 (0.45–0.81)

Abbreviations: BMI, body mass index; BP, blood pressure; CI, confidence intervals; EAF, elevated android %fat; EGF, elevated gynoid %fat; HDL-C, High-density lipoprotein-cholesterol; LDL-C, Low-density lipoprotein-cholesterol; OR, odds ratio from multivariate logistic regression analysis.

a Value is for male; if the 95% CI does not contain the value 1.0, the association is statistically significant at alpha = 0.05.

**Table 4:** Associations between android percent fat, gynoid percent fat and their joint occurrence on cardiometabolic deregulations in American men

*Independent variable*	*Cardiometabolic risk factors*				
	*Elevated glucose*	*Elevated BP*	*Elevated LDL-C*	*Elevated triglycerides*	*Low HDL-C*
	*OR (95% CI)*	*OR (95% CI)*	*OR (95% CI)*	*OR (95% CI)*	*OR (95% CI)*
EAF	0.59 (0.21–1.67)	1.21 (1.06–1.77)	0.81 (0.46–1.40)	2.41 (2.03–5.75)	1.23 (1.19–1.89)
Age	1.07 (1.03–1.11)	1.05 (1.04–1.06)	1.05 (1.04–1.07)	1.01 (0.99–1.02)	1.01 (1.00–1.03)
BMI	1.07 (0.88–1.30)	1.08 (1.05–1.24)	1.16 (1.06–1.26)	1.12 (1.03–1.23)	0.84 (0.79–0.91)
Smoking	0.77 (0.28–2.13)	1.27 (0.98–1.81)	0.79 (0.50–1.25)	1.29 (0.80–2.09)	0.95 (0.66–1.37)
Alcohol	0.53 (0.20–1.43)	1.08 (0.76–1.22)	0.97 (0.49–1.95)	1.47 (0.76–2.87)	1.70 (1.08–2.69)
EGF	0.51 (0.42–1.93)	0.86 (0.79–1.08)	0.82 (0.76–0.90)	0.97 (0.71–1.18)	0.65 (0.57–1.01)
Age	1.07 (1.06–1.11)	1.05 (1.01–1.06)	1.05 (1.03–1.08)	1.01 (0.99–1.02)	1.02 (1.01–1.04)
BMI	1.11 (1.09–1.33)	1.07 (1.02–1.25)	1.15 (1.11–1.29)	1.23 (1.11–1.37)	0.82 (0.80–0.96)
Smoking	0.73 (1.03–2.10)	1.28 (1.11–1.79)	0.83 (0.78–1.19)	1.13 (1.07–2.26)	0.96 (0.63–1.20)
Alcohol	1.94 (1.41–5.31)	0.92 (0.85–1.10)	0.99 (0.82–1.35)	0.78 (0.51–1.22)	0.57 (0.45–0.93)
EAF and EGF	1.51 (1.42–2.93)	1.48 (1.32–1.91)	1.55 (1.30–1.99)	2.47 (2.21–3.98)	1.65 (1.51–2.93)
Age	1.07 (1.06–1.09)	1.06 (1.04–1.08)	1.03 (1.03–1.06)	1.01 (0.99–1.02)	1.02 (1.01–1.03)
BMI	1.11 (1.09–1.35)	1.08 (1.05–1.21)	1.06 (1.05–1.20)	1.23 (1.14–1.27)	0.82 (0.83–0.95)
Smoking	0.73 (0.73–2.01)	1.35 (1.15–1.88)	0.83 (0.68–1.39)	1.38 (1.18–2.36)	0.96 (0.74–1.26)
Alcohol	1.94 (1.91–3.31)	1.07 (1.01–1.52)	1.00 (0.52–1.45)	0.78 (0.46–1.47)	0.57 (0.45–0.81)

Abbreviations: BMI, body mass index; BP, blood pressure; CI, confidence intervals; EAF, elevated android %fat; EGF, elevated gynoid %fat; HDL-C, High-density lipoprotein-cholesterol; LDL-C, Low-density lipoprotein-cholesterol; OR, odds ratio from multivariate logistic regression analysis; if the 95% CI does not contain the value 1.0, the association is statistically significant at alpha = 0.05.

**Table 5:** Associations between android percent fat, gynoid percent fat and their joint occurrence on cardiometabolic deregulations in American women

*Independent variable*	*Cardiometabolic risk factors*				
	*Elevated glucose*	*Elevated BP*	*Elevated LDL-C*	*Elevated triglycerides*	*Low HDL-C*
	*OR (95% CI)*	*OR (95% CI)*	*OR (95% CI)*	*OR (95% CI)*	*OR (95% CI)*
EAF	1.28 (0.33–2.44)	0.84 (0.47–1.48)	1.53 (0.74–8.59)	2.85 (0.94–3.65)	1.52 (1.29–2.93)
Age	1.22 (1.06–1.42)	1.08 (1.06–1.10)	1.03 (0.99–1.07)	1.03 (1.01–1.06)	1.01 (1.00–1.03)
BMI	1.33 (0.86–2.07)	1.00 (0.90–1.10)	0.99 (0.80–1.23)	0.98 (0.88–1.09)	0.96 (0.88–1.06)
Smoking	7.34 (0.89–9.38)	1.23 (0.72–2.09)	1.52 (0.49–4.07)	1.27 (0.68–2.36)	0.61 (0.37–1.03)
Alcohol	0.34 (0.21–2.61)	0.69 (0.38–1.24)	0.88 (0.22–3.48)	1.39 (0.63–1.47)	1.88 (0.99–3.58)
EGF	0.87 (0.72–0.93)	0.82 (0.71–0.99)	0.92 (0.78–1.10)	0.89 (0.61–2.98)	0.59 (0.52–0.91)
Age	1.29 (1.05–1.12)	1.08 (1.05–1.08)	1.04 (1.03–1.06)	1.03 (0.99–1.02)	1.01 (1.01–1.04)
BMI	1.15 (1.09–1.35)	1.01 (1.04–1.15)	1.04 (1.05–1.19)	1.15 (1.10–1.27)	0.92 (0.81–0.97)
Smoking	1.38 (1.03–3.00)	1.21 (1.11–1.79)	1.16 (0.68–1.39)	1.29 (1.00–2.36)	0.63 (0.61–1.21)
Alcohol	2.50 (1.41–5.31)	1.44 (1.05–1.50)	0.68 (0.52–1.45)	0.95 (0.46–1.47)	0.54 (0.44–0.92)
EAF and EGF	2.24 (2.01–2.93)	1.82 (1.62–1.91)	1.77 (1.50–1.89)	3.56 (2.61–3.98)	1.70 (1.49–2.93)
Age	1.22 (1.05–1.09)	1.06 (1.04–1.08)	1.03 (1.01–1.06)	1.03 (0.99–1.02)	1.01 (1.00–1.04)
BMI	1.33 (1.08–1.35)	1.08 (1.05–1.21)	0.97 (1.05–1.20)	1.02 (1.17–1.37)	0.96 (0.85–0.91)
Smoking	1.35 (1.13–3.00)	1.35 (1.15–1.88)	1.31 (0.68–1.39)	1.45 (1.18–2.36)	1.61 (0.54–1.07)
Alcohol	3.01 (1.91–5.31)	1.07 (1.01–1.52)	0.72 (0.52–1.45)	1.07 (0.46–1.47)	0.53 (0.49–0.88)

Abbreviations: BMI, body mass index; BP, blood pressure; CI, confidence intervals; EAF, elevated android %fat; EGF, elevated gynoid %fat; HDL-C, High-density lipoprotein-cholesterol; LDL-C, Low-density lipoprotein-cholesterol; OR, odds ratio from multivariate logistic regression analysis; if the 95% CI does not contain the value 1.0, the association is statistically significant at alpha = 0.05.

## References

[bib1] Karelis AD Brochu M Rabasa-Lhoret R Can we identify metabolically healthy but obese individuals (MHO) Diabetes Metab 2004 30 569 572 15671927 10.1016/s1262-3636(07)70156-8

[bib2] Boonchaya-Anant P Apovian CM Metabolically healthy obesity-does it exist Curr Atheroscler Rep 2014 16 441 25092577 10.1007/s11883-014-0441-1

[bib3] Brochu M Tchernof A Dionne IJ Sites CK Eltabbakh GH Sims EA What are the physical characteristics associated with a normal metabolic profile despite a high level of obesity in postmenopausal women J Clin Endocrinol Metab 2001 86 1020 1025 11238480 10.1210/jcem.86.3.7365

[bib4] Primeau V Coderre L Karelis AD Brochu M Lavoie ME Messier V Characterizing the profile of obese patients who are metabolically healthy Int J Obes (Lond) 2011 35 971 981 20975726 10.1038/ijo.2010.216

[bib5] Meigs JB Wilson PW Fox CS Vasan RS Nathan DM Sullivan LM Body mass index, metabolic syndrome, and risk of type 2 diabetes or cardiovascular disease J Clin Endocrinol Metab 2006 91 2906 2912 16735483 10.1210/jc.2006-0594

[bib6] Durward CM Hartman TJ Nickols-Richardson SM All-cause mortality risk of metabolically healthy obese individuals in NHANES III J Obes 2012 2012 460321 23304462 10.1155/2012/460321 PMC3523154

[bib7] Gaillard TR Schuster D Osei K Characterization of metabolically unhealthy overweight/obese African American women: significance of insulin-sensitive and insulin-resistant phenotypes Natl Med Assoc 2012 104 164 171 10.1016/s0027-9684(15)30141-3 22774383

[bib8] Hamer M Stamatakis E Metabolically healthy obesity and risk of all-cause and cardiovascular disease mortality J Clin Endocrinol Metab 2012 97 2482 2488 22508708 10.1210/jc.2011-3475 PMC3387408

[bib9] Wildman RP Muntner P Reynolds K McGinn AP Rajpathak S Wylie-Rosett J The obese without cardiometabolic risk factor clustering and the normal weight with cardiometabolic risk factor clustering: Prevalence and correlates of 2 phenotypes among the us population (NHANES 1999–2004) Arch Intern Med 2008 168 1617 1624 18695075 10.1001/archinte.168.15.1617

[bib10] Yoo HJ Hwang SY Hong HC Choi HY Seo JA Kim SG Association of metabolically abnormal but normal weight (MANW) and metabolically healthy but obese (MHO) individuals with arterial stiffness and carotid atherosclerosis Atherosclerosis 2014 234 218 223 24681911 10.1016/j.atherosclerosis.2014.02.033

[bib11] Velho S Paccaud F Waeber G Vollenweider P Marques-Vidal P Metabolically healthy obesity: different prevalences using different criteria Eur J Clin Nutr 2010 64 1043e51 20628408 10.1038/ejcn.2010.114

[bib12] Barber J Palmese L Chwastiak LA Ratliff JC Reutenauer EL Jean-Baptiste M Reliability and practicality of measuring waist circumference to monitor cardiovascular risk among community mental health center patients Community Ment Health J 2014 50 68 74 23306677 10.1007/s10597-012-9590-2

[bib13] Klein S Allison DB Heymsfield SB Kelley DE Leibel RL Nonas C Waist circumference and cardiometabolic risk: A consensus statement from shaping America's health: Association for Weight Management and Obesity Prevention; NAASO, the Obesity Society; the American Society for Nutrition; and the American Diabetes Association Obesity 2007 15 1061 1067 17495180 10.1038/oby.2007.632

[bib14] Hangartner TN Warner S Braillon P Jankowski L Shepherd J The Official Positions of the International Society for Clinical Densitometry: acquisition of dual-energy X-ray absorptiometry body composition and considerations regarding analysis and repeatability of measures J Clin Densitom 2013 16 520 536 24183641 10.1016/j.jocd.2013.08.007

[bib15] Doran DA McGeever S Collins KD Quinn C McElhone R Scott M The validity of commonly used adipose tissue body composition equations relative to dual energy X-ray absorptiometry (DXA) in gaelic games players Int J Sports Med 2014 35 95 100 23900901 10.1055/s-0033-1333693

[bib16] Eston RG Rowlands AV Charlesworth S Davies A Hoppitt T Prediction of DXA-determined whole body fat from skinfolds: importance of including skinfolds from the thigh and calf in young, healthy men and women Eur J Clin Nutr 2005 59 695 702 15798775 10.1038/sj.ejcn.1602131

[bib17] Doran DA McGeever S Collins KD Quinn C McElhone R Scott M The validity of commonly used adipose tissue body composition equations relative to dual energy X-ray absorptiometry (DXA) in gaelic games players Int J Sports Med 2014 35 95 10 23900901 10.1055/s-0033-1333693

[bib18] National Center for Health Statistics analytic guidelines [online], 2008Available at http://www.cdc.gov/nchs/data/nhanes/nhanes_general_guidelines_june_04.pdf . Accessed September 2014.

[bib19] National Center for Health Statistics, Centers for Disease Control and Prevention National Health and Nutrition Examination Survey (NHANES) Questionnaire and Exam Protocol. Available at http://www.cdc.gov/nchs/about/major/nhanes/questexam.htm .

[bib20] Executive summary of the Third Report of the National Cholesterol Education Program (NCEP) Expert Panel on Detection, Evaluation and Treatment of High Blood Cholesterol in Adults (Adult Treatment Panel III) JAMA 2001 285 2486 2497 11368702 10.1001/jama.285.19.2486

[bib21] http://www.cdc.gov/alcohol/faqs.htm#moderate Drinking. Accessed August 2014.

[bib22] Fu X Song A Zhou Y Ma X Jiao J Yang M Association of regional body fat with metabolic risks in Chinese women Public Health Nutr 2014 17 2316 2324 24148901 10.1017/S1368980013002668 PMC10282636

[bib23] Kang SM Yoon JW Ahnb HY Kim SY Lee KH Shin H Android fat depot is more closely associated with metabolic syndrome than abdominal visceral fat in elderly people PLoS One 2011 6 e27694 22096613 10.1371/journal.pone.0027694 PMC3214067

[bib24] Samsell L Regier M Walton C Cottrell L Importance of android/gynoid fat ratio in predicting metabolic and cardiovascular disease risk in normal weight as well as overweight and obese children J Obes 2014 2014 846578 25302115 10.1155/2014/846578 PMC4181515

[bib25] Stevens J Obesity, fat patterning, and cardiovascular risk Adv Exp Med Biol 1995 369 21 27 7598009 10.1007/978-1-4615-1957-7_3

[bib26] Ross R Freeman J Hudson R Janssen I Abdominal obesity, muscle composition, and insulin resistance in premenopausal women J Clin Endocrinol Metab 2002 87 5044 5051 12414870 10.1210/jc.2002-020570

[bib27] Blouin K Boivin A Tchernof A Androgens and body fat distribution J Steroid Biochem Mol Biol 2008 108 272 280 17945484 10.1016/j.jsbmb.2007.09.001

[bib28] Björntorp P The regulation of adipose tissue distribution in humans Int J Obes Relat Metab Disord 1996 20 291 302 8680455

[bib29] Staiano AE Katzmarzyk PT Ethnic and sex differences in body fat and visceral and subcutaneous adiposity in children and adolescents Int J Obes (Lond) 2012 36 1261 1269 22710928 10.1038/ijo.2012.95 PMC4129655

[bib30] Fu X Song A Zhou Y Ma X Jiao J Yang M Association of regional body fat with metabolic risks in Chinese women Public Health Nutr 2013 22 1 9 10.1017/S1368980013002668 PMC10282636 24148901

[bib31] De Larochellière E Côté J Gilbert G Bibeau K Ross MK Dion-Roy V Visceral/epicardial adiposity in nonobese and apparently healthy young adults: association with the cardiometabolic profile Atherosclerosis 2014 234 23 29 24589564 10.1016/j.atherosclerosis.2014.01.053

[bib32] Aucouturier J Meyer M Thivel D Taillardat M Duché P Effect of android to gynoid fat ratio on insulin resistance in obese youth Arch Pediatr Adolesc Med 2009 163 826 831 19736336 10.1001/archpediatrics.2009.148

[bib33] Peppa M Koliaki C Hadjidakis DI Garoflos E Papaefstathiou A Katsilambros N Regional fat distribution and cardiometabolic risk in healthy postmenopausal women Eur J Intern Med 2013 24 824 831 24169066 10.1016/j.ejim.2013.07.001

[bib34] Kang SM Yoon JW Ahn HY Kim SY Lee KH Shin H Android fat depot is more closely associated with metabolic syndrome than abdominal visceral fat in elderly people PLoS One 2011 6 e27694 22096613 10.1371/journal.pone.0027694 PMC3214067

[bib35] Landis JR Lepkowski JM Eklund SA Stehouwer SA A statistical methodology for analyzing data from a complex survey: the first National Health and Nutrition Examination Survey Vital Health Stat 2 1982 92 1 52 7179756

[bib36] Barreira TV Broyles ST Gupta AK Katzmarzyk PT Relationship of anthropometric indices to abdominal and total body fat in youth: sex and race differences Obesity (Silver Spring) 2014 22 1345 1350 24493150 10.1002/oby.20714 PMC4008658

